# Cortical Short-Range Fiber Connectivity and Its Association With Deep Brain White Matter Hyperintensities in Older Diabetic People With Low Serum Vitamin B_12_

**DOI:** 10.3389/fnagi.2022.754997

**Published:** 2022-03-25

**Authors:** Kai Liu, Xiaopeng Wang, Teng Zhang, Wei Wang, Ruohan Li, Li Lu, Yanjia Deng, Kai Xu, Timothy Kwok

**Affiliations:** ^1^Department of Radiology, Affiliated Hospital of Xuzhou Medical University, Xuzhou Medical University, Xuzhou, China; ^2^Department of Neurology, Affiliated Hospital of Xuzhou Medical University, Xuzhou, China; ^3^Department of Nuclear Medicine and PET-CT Center, The Second Hospital of Zhejiang University School of Medicine, Hangzhou, China; ^4^School of Medical Imaging, Xuzhou Medical University, Xuzhou, China; ^5^Department of Medicine and Therapeutics, The Chinese University of Hong Kong, Shatin, Hong Kong SAR, China

**Keywords:** vitamin B_12_, short-range fiber connectivity, diffusion tensor imaging, white matter hyperintensities, diabetes mellitus

## Abstract

Although previous studies have indicated that older people with diabetes mellitus (DM) had an approximately two times larger white matter hyperintensity (WMH) load than those without DM, the influence of WMHs on cognition is uncertain and inconsistent in the literature. It is unclear whether the short-range fibers in the juxtacortical region, traditionally considered to be spared from WMH pathology, are enhanced as an adaptive response to deep WM degeneration in older diabetic people with normal cognition. Moreover, the specific effect of vitamin B_12_ deficiency, commonly accompanied by DM, remains to be investigated. This study implemented a specialized analysis of the superficial cortical short-range fiber connectivity density (SFiCD) based on a data-driven framework in 70 older individuals with DM and low serum vitamin B_12_. Moreover, the effects of time and vitamin B_12_ supplementation were assessed based on a randomized placebo-controlled trial in 59 individuals. The results demonstrated a higher SFiCD in diabetic individuals with a higher deep WMH load. Additionally, a significant interaction between DWMH load and homocysteine on SFiCD was found. During the 27-month follow-up period, a longitudinal increase in the SFiCD was observed in the bilateral frontal cortices. However, the observed longitudinal SFiCD change was not dependent on vitamin B_12_ supplementation; thus, the specific reason for the longitudinal cortical short fiber densification may need further study. Overall, these findings may help us better understand the neurobiology of brain plasticity in older patients with DM, as well as the interplay among DM, WMH, and vitamin B_12_ deficiency.

## Introduction

A previous study indicated that older people with diabetes mellitus (DM) had an approximately two times larger white matter hyperintensity (WMH) load than those without DM (Vergoossen et al., 2021). However, the relationship between WMHs and cognitive decline in older people with DM is uncertain and inconsistent among studies. For instance, some findings support a direct relationship between WMH load and cognitive impairment in older diabetic people (Biessels et al., 2008; Callisaya et al., 2019), whereas other results have shown only slight or insignificant cognitive consequences (Reijmer et al., 2013; Vergoossen et al., 2021). To explain this discrepancy, some cognitive reserve or compensatory mechanisms are hypothesized to partly preserve normal brain functioning against WMHs and thus confound the association between WMH burden and cognitive consequences (Galluzzi et al., 2008; Mortamais et al., 2013). However, these hypotheses have yet to be tested thoroughly.

The effect of WMHs on cognition is mediated by the disconnection of brain white matter (WM) fibers that normally connect the brain cortex (Galluzzi et al., 2008; Griebe et al., 2011). In particular, the long-distance fiber connections embedded in deep brain WM regions are usually the prime target of WMH lesions (i.e., deep WMHs, DWMHs) (Griebe et al., 2011; Jacobs et al., 2012; Reijmer et al., 2015). In contrast, the association between WMHs and short-range fiber connections that occupy the peripheral WM regions adjacent to the cortex has not been fully investigated. Unlike the deep WM with its solitary blood supply, the juxtacortical/subcortical WM, classically consisting of subcortical U-fibers (Pron and Deruelle, 2021), is supplied by both long penetrating medullary vessels and adjacent short cortical vessels (Moody et al., 1990; Kim et al., 2008). Thus, superficial subcortical short-range fibers are considered to be mostly spared from WMH pathologies (Kim et al., 2008) and usually appear as normal-appearing white matter (NAWM) regions on conventional MRI.

Nevertheless, it is worth noting that the changed integrity of the NAWM on diffusion tensor imaging (DTI) scans in individuals with WMHs has aroused much interest in recent years (Maniega et al., 2015; Tuladhar et al., 2015). The results of these studies have shown a strong relationship between abnormal integrity in the NAWM and the severity of WMHs (Maniega et al., 2015). More importantly, Tuladhar et al. (2015) found that most of the significant associations between WM integrity and cognitive performance were found in NAWM instead of WMH lesion areas. Similarly, Reijmer et al. (2013) found that the association between cognitive performance and whole-brain WM microstructural properties was not dependent on the WMH load in diabetic individuals. Therefore, these findings highlight the active role of the NAWM in the brain under a WMH burden and may also lead us to further focus on the alteration of seemingly healthy cortical short-range fibers in older people with DM.

Previous DTI studies specific to the superficial WM are largely dependent on and confined within a prior-selected brain region of interest (ROI) (Gao et al., 2014; d’Albis et al., 2018). Thus, the results may be less spatially detailed and easily biased by the definition of the ROI. In this study, to achieve a specialized and unbiased evaluation of cortical short-range fiber connectivity, a data-driven exploratory analysis framework was designed by combining cortical surface reconstruction (Dale et al., 1999; Fischl et al., 1999) and diffusion tractography techniques (Mori and van Zijl, 2002), namely, short-range fiber connectivity density (SFiCD). With this approach, we aimed to investigate the specific pattern of change in cortical short-range fiber connectivity as well as its association with DWMHs in older people with DM.

Interestingly, based on the existing literature, two competing processes are hypothesized. First, based on previous findings from NAWM studies, the superficial short-range connectivity of the cortex is hypothesized to decrease as the DWMH burden in older diabetic individuals increases, reflecting a global WM degeneration shared by both the superficial and the deep brain (Griebe et al., 2011; Reijmer et al., 2013; Maniega et al., 2015). Second, in response to the neural disconnection effect caused by WMHs in the deep brain, it is possible that alternative neural pathways (or “latent” connections) may be recruited among the cortical short fibers in the superficial brain, which has higher plasticity and thus leads to enhanced short connectivities to preserve normal cognitive functions in older people without dementia (Galluzzi et al., 2008; Reijmer et al., 2015). Moreover, it is known that both DM and aging are significant risk factors for vitamin B_12_ deficiency, which leads to vitamin B_12_ deficiency being common in older people with DM (Tavares Bello et al., 2017). In fact, vitamin B_12_ deficiency causes the accumulation of homocysteine in the brain, which ultimately inhibits the myelination of WM fibers (Vafai and Stock, 2002; Popp et al., 2009). Therefore, in this study, we were also interested in whether vitamin B_12_ supplementation and serum homocysteine, as factors related to myelination of WM fibers, play a role in the modification of short-range fibers. We believe the findings will help to better elucidate how brain WM connectivities reorganize against WMH pathologies in older people with DM.

## Materials and Methods

### Participants

The subjects of this study were a subgroup from a randomized placebo-controlled trial of vitamin B_12_ supplementation in older diabetic people who had undergone MR scans with DTI sequence; this trial has been described elsewhere (Kwok et al., 2017). In brief, 271 older medical outpatients with DM and borderline low serum vitamin B_12_ (150–300 pmol/L) were randomly assigned to receive oral vitamin B_12_ supplements or placebo for 27 months. Blood samples were taken after an overnight fast and were then analyzed for serum concentrations of homocysteine and methylmalonic acid (MMA). Cognitive function was measured by the scores of the clinical dementia rating scale (CDR) (Lam et al., 2008) and neurocognitive test battery (NTB) (Shen et al., 2014). The tests of the NTB included the Controlled Oral Word Association Test (COWAT) and the Category Fluency Test (CFT) for executive function; “Detection,” a test of the Simple Reaction Time test (SRT) and “Identification,” a Choice Reaction Time test (CRT) for psychomotor speed and attention; the International Shopping List Test (ISLT) for verbal memory; and the Continuous Paired Associates Learning (CPAL) for episodic visual memory. Then, the *Z* score of each test was calculated by subtracting the baseline mean score and then dividing the baseline standard deviation. Finally, the executive function, psychomotor processing speed, and memory scores were calculated as the average *Z* score of the COWAT and CFT, the average Z score of the SRT and CRT, and the average Z score of the CPAL and ISLT, respectively. In addition, the CDR score (sum of boxes) was used as an overall assessment of global cognitive status and for screening. All measurements were repeated at month 27. All participants gave informed written consent, and ethical approval was obtained from the medical ethics committee of our institution (Clinical trial registry of the US: NCT02457507).

In this study, we aimed to examine the potential role of cortical short-range fibers in older people with DM with normal cognition. Therefore, only participants with a CDR score of ≤ 0.5 were included. Moreover, individuals with infarct lesions on brain MRI were excluded to limit the confounding effect of stroke. MR images from each individual were evaluated by an experienced radiologist and a neurologist for consensus. Assessment of brain WMH load was performed in accordance with the standard of the Fazekas scale (Fazekas et al., 2002). To explore the alteration in the cortical short fiber connectivity in relation to DWMHs in diabetic individuals, the participants were divided into a low-load DWMH group and a high-load DWMH group according to previous studies (Fazekas et al., 2002; Patti et al., 2016). Specifically, participants with a Fazekas score of 0 or 1 (no or punctate foci) and those with a Fazekas score of 2 or 3 (beginnings of focus confluence or large confluent areas) in the deep brain WM were defined as the low-load DWMH group and high-load DWMH group, respectively.

Other details regarding the exclusion of participants were described in our previous study (Kwok et al., 2017).

### MRI Acquisition

MR scans were performed for each participant with a 3T MR scanner (Achieva, Philips Medical Systems, Best, The Netherlands) and an 8-channel head coil. Three-dimensional T1-weighted images (3D-T1WI) were acquired using a turbo field echo (TFE) sequence with the following parameters: repetition time (TR)/echo time (TE) = 6.8/3.1 ms, flip angle = 9°, field of view (FOV) = 256 × 256 mm, and voxel size = 1.2 × 1.0 × 1.0 mm. DTI scans were acquired using an echo planar imaging (EPI) sequence with the following parameters: TR/TE = 8568.7/60.0 ms, flip angle = 90°, FOV = 224 × 224 mm, voxel size = 1.0 × 1.0 × 2.0 mm, *b* = 1,000 s/mm^2^, number of diffusion weighting directions = 32, and one *b*_0_ volume. Moreover, fluid-attenuated inversion-recovery (FLAIR) images were collected for rating the WMHs using an inversion recovery sequence with the following parameters: TR/TE/inversion time (TI) = 11,000/125/2,800 ms, flip angle = 90°, FOV = 230 × 230 mm, pixel size = 0.33 × 0.33 mm, and thickness = 5 mm.

### Preprocessing

The 3D-T1WI anatomical images were preprocessed following the standard pipeline of FreeSurfer (version 6.0.0, surfer.nmr.mgh.harvard.edu). Finally, with all the FreeSurfer procedures, a GM–WM interface was constructed (Fischl et al., 2002). This interface corresponded to the surface where the connections between the cortical GM ribbon and subcortical WM fibers were established, and the calculation of fiber density was performed on this surface in the following steps. See *Preprocessing of 3D-T1WI* in Supplementary Appendix for more details. Preprocessing of diffusion images followed the standard steps of the FSL tool.^1^ Images were corrected for head motion and eddy current, and then the brain tissue was extracted using the BET toolbox.

### Extraction of Short-Range Fiber Tracks

Based on the preprocessed diffusion images, WM tractography was generated using a deterministic fiber tracking approach (streamline algorithm) utilizing the DSI studio tool (Yeh et al., 2013). The parameters of the fiber tracking were as follows: fractional anisotropy (FA) threshold = 0.14, turning angle threshold = 35°, step size = 0.5, smoothing = 0.5, and seed number = 1/voxel. Notably, for the short-range connectivity measurement of the brain cortex, a fiber length constraint was decided based on previous evidence to extract the cortical short-range fibers while fiber tracking was being performed. First, according to Sundaram et al. (2008), the mean lengths of the long and short white matter tracts were estimated to be 118.2 and 35.3 mm, respectively, based on diffusion data. Second, Shukla et al. (2011) adopted a cutoff value of 65 mm to extract short white matter tracts. Third, our preliminary length-spectrum test showed that typical U-fibers appeared in the range of 10–15 mm (fibers < 10 mm in length were mostly false tracts; see Supplementary Figure 1). Meanwhile, U-fibers began to disappear in the range of 65–70 mm. Taken together, the final fiber length constraint of 10–65 mm was chosen for tractography construction.

### Calculation of Short-Range Fiber Connectivity Density

**FIGURE 1:**
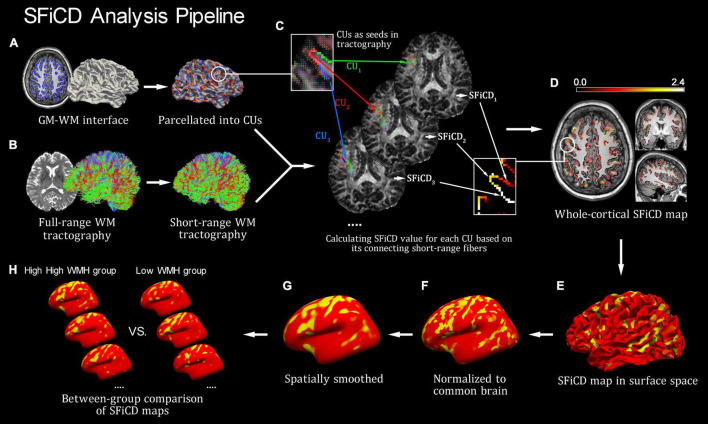
Introduction of the short-range fiber connectivity density (SFiCD) mapping framework. **(A)** Cortical parcellation and cortical units (CUs). **(B)** Construction of short-range fiber tractography. **(C)** Calculation of SFiCD value for each CU. **(D)** Whole-cortical SFiCD map in volume space. **(E)** SFiCD map projected to surface space. **(F)** Spatial normalization of SFiCD map. **(G)** Spatial smooth of SFiCD map. **(H)** Group-level vertex-wise statistical analysis of cortical SFiCD.

The SFiCD was calculated by essentially following our previously established analysis pipeline of fiber connectivity density (FiCD), which realized a data-driven approach for whole-cortical connectivity calculation and comparison (Liu et al., 2017). The difference is that short-range WM fiber tractography was used instead, thus realizing a special FiCD calculation for short-range fibers (i.e., SFiCD). The basic procedures are as follows. First, the whole-cortical GM–WM interface created during preprocessing was parcellated into one thousand small surface patches (termed cortical units, CUs) (Figure 1A). Then, the subcortical voxel layer under each patch was located and transformed into tractography space as a segmentation mask. Second, each CU mask was used as a seed to track its connecting short-range fibers in the constructed short-range WM tractography (constructed in previous steps) (Figure 1B). Then, the SFiCD was calculated as the sum of the fractional anisotropy (FA) values of all the short-range fibers connecting to a single CU and then divided by the volume of the CU (Figure 1C):


$$S\,F\,i\,C\,D=\frac{\sum_{f\in N}F\,A_{f}}{V_{C\,U}}$$

Here, for a single CU, *N* represents the total number of short-range fiber tracts connecting to it; *f* stands for a generic connecting fiber; *FA*_*f*_ represents the mean FA value for *f*; and *V*_*CU*_ represents the volume of this CU. Thereafter, by assigning the calculated SFiCD value back to each corresponding CU, a whole-cortical SFiCD map (Figures 1D,E) was constructed. Third, for group-level comparison, the cortical SFiCD map of each subject was spatially normalized to a common cortical surface (Figure 1F) and then smoothed with a 15 mm full-width at half-maximum (FWHM) Gaussian kernel to reduce the noise caused by normalization (Figure 1G). Here, to ensure an accurate alignment of the gyral structures among individuals, an intersubject surface registration was performed by registering the cortical surface with the depth of the gyri and sulci normalized to an averaged spherical surface (Fischl et al., 1999). Finally, a vertexwise statistical comparison of the cortical SFiCD was performed between the high-load and low-load DWMH groups (Figure 1H).

### Statistical Analysis

Differences between the high-load and low-load DWMH groups in terms of age, education, total intracranial volume, total WM volume, WMH volume [automatic segmentation results created based on 3D-T1WI using FreeSurfer (Dadar et al., 2018; Jung et al., 2021)], serum levels of homocysteine and MMA, and the CDR (sum of boxes) and NTB scores were evaluated with a two-sample *t*-test (or Mann–Whitney *U*-test for non-normally distributed data). Between-group differences in sex were assessed with the χ^2^-test.

Vertexwise statistical analyses of cortical SFiCD were performed using a general linear model (GLM) with a significance threshold at vertex-level uncorrected *P* < 0.01 and cluster-level Monte Carlo null-*Z* simulation-corrected *P* < 0.01 (10,000 iterations). Differences in cortical SFiCD between the high-load and low-load DWMH groups and the interaction effect of “DWMH × homocysteine” on cortical SFiCD were assessed using a “different-offset, different-slope” (DODS) design. Age, sex, education, and total intracranial volume were added as nuisance covariates to regress out their confounding effect. Moreover, the mean SFiCD value of each cluster with significant between-group differences from the vertexwise analyses was extracted and correlated to the CDR and NTB scores using the partial correlation test (controlling for age, sex, education, and total intracranial volume). A significance threshold of *P* < 0.05 was used.

The longitudinal SFiCD change was calculated as the “rate” (i.e., change in SFiCD value per month) for each vertex, and then within-group and between-group (active group vs. placebo group and high-load DWMH group vs. low-load DWMH group) statistical analyses were performed using the GLM. Here, it is worth noting that the two between-group comparisons for SFiCD rate were essentially assessments of the interaction effects of “Time × DWMH” and “Time × vitamin B_12_ supplementation” on the SFiCD, since the factor of “Time” was taken into account by using “SFiCD rate.” Similarly, for the between-group analyses of the rate of change in the SFiCD, age, sex, education, and total intracranial volume were added as nuisance covariates.

### Validation Analysis

To ensure that the observed SFiCD variations were not dependent on the effect of confounding factors, we assessed the relationships between the resulting SFiCD value(s) and several confounding factors, including age, education, total intracranial volume, sex, and the use of metformin and aspirin. Here, for dichotomous confounding factors, between-group comparisons (instead of correlations) were performed to assess the differences in the SFiCD driven by these factors. Then, for the possible impact of dynamic changes in WMH volume during the 27-month follow-up, we further assessed the correlation between the longitudinal change in the WMH segmentation volume and the change in the SFiCD. Finally, for the longitudinal analysis, which included multiple factors of interest, a linear regression was performed by including supplementation with vitamin B_12_, baseline DWMH load, longitudinal change in WMH volume, homocysteine level, age, sex, education, total intracranial volume, and the use of metformin and aspirin as independent factors to assess their contribution to longitudinal SFiCD changes.

## Results

### Demographic Variables

In total, 32 and 38 participants were included in the high-load and low-load DWMH groups, respectively. No significant between-group differences in age, sex, education, total intracranial volume, total cerebral WM volume, or CDR (sum of boxes) or NTB scores were found. Unbiased automatic segmentations of the WMHs (created by FreeSurfer) from each individual brain were normalized to the standard brain to show the spatial distribution of the lesions (Supplementary Figure 2). The segmentation volume of the WMHs was significantly larger in the high-load DWMH group than in the low-load DWMH group (*P* < 0.001). Moreover, serum homocysteine and MMA levels did not significantly differ between the two groups (*P* = 0.153). See Table 1 for more detailed statistics.

**TABLE 1:** Comparison of demographic data between the high-load and low-load deep white matter hyperintensity (DWMH) groups.

	High-load DWMH group (*n* = 32)	Low-load DWMH group (*n* = 38)	Statistics	*P*-value
Age (years)	73.5 (69–83)	74 (69–83)	*Z* = –0.148	0.882
Sex (female/male)	15/17	14/24	χ^2^ = 0.721	0.396
Education (years)	5.25 (0–15)	4 (0–18.5)	*Z* = –0.532	0.594
Total intracranial volume (×10^5^ mm^3^)	14.52 (11.49–17.71)	14.74 (9.41–18.12)	*Z* = –0.171	0.864
Cerebral white matter volume (×10^5^ mm^3^)	3.99 ± 0.40	3.95 ± 0.52	*T* = –0.356	0.723
WMH segmentation volume (×10^3^ mm^3^)	6.68 (2.37–36.14)	2.98 (1.07–40.04)	*Z* = –4.456	< 0.001*
Serum homocysteine (μmol/L)	16.74 (8.21–32.68)	17.13 (10.35–50.92)	*Z* = –0.802	0.423
Serum MMA (μmol/L)	0.23 (0.10–0.78)	0.18 (0.10–0.82)	*Z* = –1.428	0.153
CDR sum	0.5 (0–3)	0.5 (0–3)	*Z* = –0.351	0.726
Executive function	0.08 ± 0.78	0.21 ± 0.99	*T* = 0.603	0.549
Psychomotor speed	0.04 ± 0.88	0.06 ± 0.68	*T* = 0.085	0.932
Memory	0.10 ± 0.80	0.15 ± 0.91	*T* = 0.212	0.833

*Statistically significant; normally and non-normally distributed data are described as the mean ± standard deviation and median (range), respectively; CDR, clinical dementia rating.

### Vertexwise Short-Range Fiber Connectivity Density Comparison Between Low-Load and High-Load DWMH Groups

**FIGURE 2:**
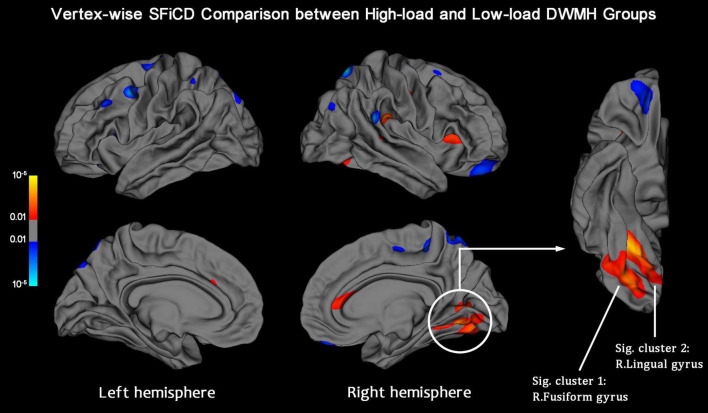
Vertexwise SFiCD comparison between the low-load and high-load DWMH groups. The statistical map is thresholded with a vertex-level *P* < 0.01. The cluster that survived Monte Carlo simulation correction (cluster-level *P* < 0.01) is further marked with a circle. Compared with the low-load DWMH group, the high-load DWMH group showed a significantly increased SFiCD in the right fusiform gyrus (Sig. cluster 1) and right lingual gyrus (Sig. cluster 2).

Cortical SFiCD maps of the right and left hemispheres were constructed for each participant, and the average SFiCD maps of the high-load and low-load DWMH groups are shown in Supplementary Figure 3. Vertexwise between-group comparisons of SFiCD maps at baseline were assessed using the GLM with a DODS design. Compared with the low-load DWMH group, the high-load DWMH group showed a significantly increased SFiCD in the right fusiform gyrus and right lingual gyrus (vertex-level *P* < 0.01 with Monte Carlo simulation-corrected cluster size *P* < 0.01) (Figure 2). On the other hand, no cluster in which the SFiCD decreased survived correction. In addition to the between-group comparison, vertexwise correlation between the SFiCD and segmentation volume of the WMHs was assessed. The result was consistent with the between-group comparison in that the cortical region corresponding to the right fusiform gyrus and lingual gyrus was noted to have a significant positive correlation (Supplementary Figure 4).

### Interaction Effect of “DWMH × Homocysteine” on the Cortical Short-Range Fiber Connectivity Density

**FIGURE 3:**
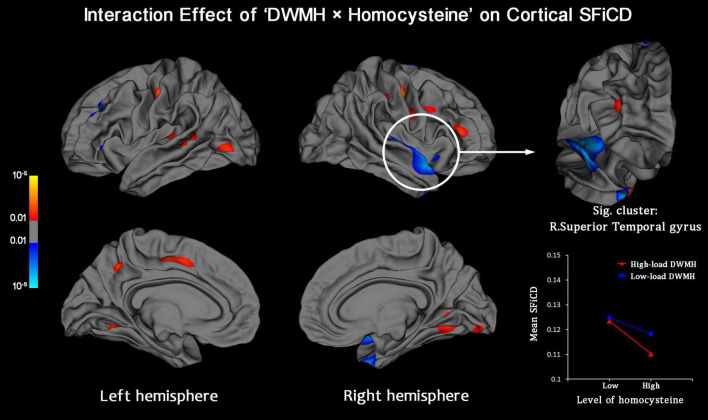
Vertexwise analysis of the interaction effect between the factors “DWMH” and “Homocysteine” on the cortical SFiCD. The statistical map is thresholded with a vertex-level *P* < 0.01. The cluster that survived Monte Carlo simulation correction (cluster-level *P* < 0.01) is further marked with a circle. The right superior/transverse temporal gyrus showed a significant interaction effect.

Vertexwise statistical analysis of the “DWMH × homocysteine” interaction effect on the cortical SFiCD was performed to determine whether the influence of DWMHs on the cortical SFiCD was dependent on the level of homocysteine. Here, it should be noted that the homocysteine data followed a non-normal distribution (Kolmogorov–Smirnov test *P* = 0.002). Therefore, to avoid bias caused by extreme values and ensure the stability of the GLM analysis, the level of serum homocysteine was not added as a continuous variable but was binarized as > or < the median level across all the included participants. A significant interaction was found in the right superior/transverse temporal gyrus (Monte Carlo simulation-corrected cluster size *P* < 0.01) (Figure 3). Specifically, a more remarkable association between higher homocysteine levels and lower SFiCD in the right superior/transverse temporal region was noted in the high-load DWMH group than in the low-load DWMH group. However, the mean SFiCD in the superior/transverse temporal gyrus did not significantly differ between the high-load and low-load DWMH groups (*P* = 0.836).

### Correlation Between Cognitive Performance and Short-Range Fiber Connectivity Density in Significant Regions

The mean SFiCD values of the two clusters with significant between-group differences, as mentioned above, were extracted and correlated to the CDR (sum of boxes) and NTB scores (Supplementary Table 1). The results showed that executive function was negatively correlated with the SFiCD in the right fusiform gyrus (*r* = –0.321, *P* = 0.018).

### Longitudinal Short-Range Fiber Connectivity Density Changes at the 27-Month Follow-Up

To further observe the longitudinal pattern of change in cortical short-range fibers during the aging process and to investigate the effect of vitamin B_12_ supplementation, a randomized placebo-controlled trial with a 27-month follow-up was performed. Ultimately, among all 70 participants enrolled for baseline analysis, 59 who finished the follow-up and underwent a second MR scan were included in the longitudinal analysis (10 subjects who failed to provide full data at 27 months and one who failed the visual inspection as part of the FreeSurfer pipeline were excluded). Age, sex, education, total intracranial volume, total cerebral WM volume, and cognitive scores were matched between the active and placebo treatment groups (Supplementary Table 2) and between the high-load and low-load DWMH groups (Supplementary Table 3). The segmentation volume of the WMHs was significantly larger in the high-load DWMH group than in the low-load DWMH group (*P* < 0.001). After the 27-month trial, a significantly larger decrease in serum MMA and homocysteine was observed in the active group than in the placebo group (both *P*s < 0.001, see Supplementary Table 2), indicative of successful supplementation with vitamin B_12_.

**FIGURE 4:**
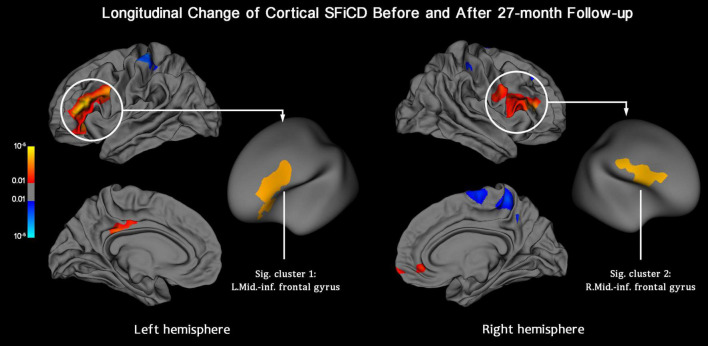
Longitudinal change in the SFiCD after the 27-month follow-up period (within-group analysis). The statistical map is thresholded with a vertex-level *P* < 0.01. The clusters that survived Monte Carlo simulation correction (cluster-level *P* < 0.01) are further marked with circles. After 27 months, the cortical SFiCD was significantly increased in the bilateral middle-inferior frontal cortices (Sig. clusters 1 and 2).

Within-group vertexwise analysis of the factor “Time,” conducted by including all 59 participants, revealed a significant SFiCD increase in the bilateral middle-inferior frontal cortices (vertex-level *P* < 0.01 with Monte Carlo simulation-corrected cluster size *P* < 0.01) (Figure 4). However, the longitudinal rate of change of the SFiCD did not significantly differ between the active and placebo groups (Supplementary Figure 5) or between the high-load and low-load DWMH groups during the 27-month follow-up (Supplementary Figure 6).

For ease of interpretation, the results are separately summarized for the cross-sectional and longitudinal parts of the study in Table 2.

**TABLE 2:** Overview of the results of the analyses of cross-sectional (Part A) and longitudinal data (Part B).

	Design (*n*)	Major type of statistic	Major factor of interest	Confounders	Major outcome	Interaction with other factor(s)?
Part A	Case control (32 vs. 38)	Between-group	DWMH load	Age, sex, education, TIV, metformin, aspirin	Higher SFiCD in high-load DWMH group	Homocysteine? Y
Part B	Cohort (59)	Repeated measure, within-group	Time	Age, sex, education, TIV, metformin, aspirin	Increased SFiCD after 27 months	VB_12_ supplementation? N DWMH load? N

*DWMH, deep white matter hyperintensity; TIV, total intracranial volume; SFiCD, short-range fiber connectivity density; VB_12_, vitamin B_12_.*

### Validation Analysis

The results of the validation analysis are provided in Supplementary Appendix and Supplementary Table 4.

## Discussion

In this study, we explored cortical short-range connectivity in older people with DM under different DWMH loads as well as its longitudinal changes under the effects of vitamin B_12_ supplementation. According to the results, the short-range fiber connectivity was higher under a higher DWMH load and increased during a 27-month follow-up. Taken together, the findings may support our hypothesis of enhanced cortical short fiber recruitment in older people with DM and without dementia.

In the cross-sectional analysis, the finding of an increased SFiCD in the high-load DWMH group was in line with the concept of a reserve mechanism proposed by previous studies said to compensate for the damage associated with WMHs and maintain a normal level of brain function (Galluzzi et al., 2008; Stern, 2012). Compared with long-range WM fibers, cortical short-range fibers preserve a less myelinated structure to facilitate higher plasticity (McKerracher et al., 1994; McGee et al., 2005; Catani et al., 2012) and were found to be more relevant to higher-level cognitive performance (e.g., social cognition; d’Albis et al., 2018). In patients with multiple sclerosis, short-range connections showed common but less severe damage than long-range fibers (Meijer et al., 2020). In comparison, for individuals with WMHs where WM impairment is less severe, previous studies have demonstrated that a higher WMH load was related to increased local connectivities and thus possibly contributed to the maintenance of normal brain function (Kim et al., 2015; Tuladhar et al., 2015). More importantly, specific to diabetic individuals, Laura W.M. Vergoossen et al. (2021) recently found that a larger WMH was associated with stronger local efficiency but not global efficiency, which is consistent with our findings and supports the hypothesis of increased regional interactions among neighboring regions driven by WMH pathology.

Specific to the cortical region of the fusiform gyrus, we found a negative association between its SFiCD and executive function. Abundant studies have shown coactivation of the fusiform gyrus and inferior frontal lobe when performing executive tasks (Utevsky and Smith, 2017; Díaz-Gutiérrez et al., 2020), supporting the involvement of the fusiform gyrus in executive function (especially in cognitive control) (Weiner and Zilles, 2016). Previous studies have also demonstrated that cognitive control function strongly predicts verbal fluency and category fluency performance (Bizzozero et al., 2013; Weiner and Zilles, 2016), which are the key components of the COWAT and CFT (i.e., executive domain of the NTB battery). A direct neural connection between the fusiform gyrus and inferior frontal cortex is known to exist in the inferior longitudinal fasciculus (ILF), which is a classic long-range association fiber bundle vulnerable to white matter pathologies in older people (Fjell et al., 2017). More importantly, a disconnected ILF in older people was also related to impaired cognitive control (Fjell et al., 2017). In our data, the participants with a higher DWMH load simultaneously showed well-preserved executive function (*P* = 0.549) and a larger SFiCD in the fusiform gyrus than low-load individuals. Therefore, the association between executive function and increased SFiCD in the fusiform gyrus may be interpreted as an adaptive response compensating for the disconnection of the ILF. In line with the negative correlation, this adaptive response, as observed in the fusiform gyrus, is possibly more significant in individuals who show a higher risk for executive dysfunction. However, this compensatory effect mediated by the SFiCD is speculative and should be interpreted with caution, especially given the lack of a patient group showing impaired executive function to demonstrate a failed compensation process.

In this study, we also assessed the interaction effect of the WMH load and serum homocysteine level on cortical short-range connectivity. Accumulated homocysteine results in endothelial dysfunction (Wu et al., 2019) by reducing the ability of endothelial cells to regulate vascular tone and inducing the stimulation of inflammatory cascades in endothelial cells (Smith and Refsum, 2021). Thus, elevated homocysteine is considered a risk factor for atherosclerosis and vessel diseases (Wang et al., 2003; Thampi et al., 2008), including white matter damage (Smith and Refsum, 2021). Specific to the short fibers, our results revealed a significant interaction effect of homocysteine × DWMH on the cortical SFiCD. However, we also noted that the homocysteine level *per se* was unable to directly induce significant differences in the SFiCD. Additionally, according to the randomized placebo-controlled trial, vitamin B_12_ supplementation, although significantly suppressing serum homocysteine, did not induce a significant effect on the short-range fiber connections over 27 months. Therefore, these findings may indicate that accumulated homocysteine and related vasculopathy possibly have some conditional influence on the cortical short-range fibers in older diabetic individuals with DWMHs, although the effect is limited and probably not independent. This may also explain why the observed cortical short fiber densification cannot be reversed by vitamin B_12_ supplementation, casting doubt on the validity of vitamin B_12_ supplementation in older people with DM for improving short-range fiber connections.

However, the longitudinal SFiCD increase probably cannot be attributed to an irreversible white matter injury (induced either by initial low vitamin B_12_ or by DM) given the insignificant interaction between time and DWMH. Geometrically, it is possible that the wrinkling process of the brain cortex driven by cortical atrophy during aging may compact the neighboring subcortical WM and thus lead to a relative densification of subcortical fibers. However, this hypothesis cannot be well supported by our results based on the insignificant correlation between the longitudinal changes in the SFiCD and cortical surface area. For another possible reason, previous evidence has consistently demonstrated that the prefrontal cortices are more vulnerable to an age-related decline (Barrick et al., 2010; Brickman et al., 2012). Furthermore, a cognitive training experiment in older people indicated that the integrity of the frontal WM was predictive of the benefit from cognitive training (de Lange et al., 2016). Therefore, the longitudinal SFiCD increase could be attributed to the aging process *per se* (i.e., an independent factor) and interpreted as an “anti-age” response that maintains relatively normal cognitive functions in older people. In line with this, this response was found to be most remarkable in the prefrontal cortices, which have higher plasticity and are more sensitive to age-related decline. However, conclusions cannot be safely drawn without a control group with normal serum vitamin B_12_ levels, and thus, further studies are still encouraged to uncover the specific mechanism.

The strengths of this study include its longitudinal design with repeated MRI, introduction of a clinical trial cohort, specialized methodology for cortical short-range fiber measurement, etc. However, several limitations of this study should be noted. First, the sample size was comparatively small, and consequently, the lack of a replication cohort might limit the generalizability of the results. More importantly, the lack of a patient group with cognitive impairment in this study may impede a solid conclusion about the underlying compensatory mechanism driven by cortical short-range fibers. Thus, large population-based observations of the short-range fibers in older people with a wider range of cognitive capability are still highly encouraged. Second, comorbidities of DM (e.g., hypothyroidism) in older people vary and are difficult to fully address in the validation analysis. Additionally, the possibility that SFiCD differences may appear after 27 months cannot be excluded. Thus, the present findings should be interpreted cautiously given these confounders. Finally, the metric of short-range connectivity, SFiCD, although established based on existing techniques and parameters, was proposed for the first time. Thus, its neurobiological relevance and the interpretation of its results need further study.

To conclude, based on a data-driven analysis framework (i.e., SFiCD analysis), this study realized an evaluation of superficial cortical short-range fiber connectivity in older people with DM and low serum vitamin B_12_ under the effects of DWMHs and vitamin B_12_ supplementation. The results demonstrated higher short-range fiber connectivity in the fusiform and lingual cortices in diabetic individuals with higher DWMH loads. Moreover, a significant interaction between the DWMH load and homocysteine level on the SFiCD was found, implying that the DWMH-driven effect on cortical short-range fibers is possibly mediated by accumulated homocysteine. During the 27-month follow-up period, a longitudinal increase in the SFiCD was observed in the bilateral frontal cortices. However, the observed longitudinal SFiCD change was not dependent on vitamin B_12_ supplementation; thus, the specific reason for the longitudinal cortical short fiber densification may need further study. Overall, these findings may help us better understand the neurobiology of brain plasticity in older patients with DM, as well as the interplay among DM, WMH, and vitamin B_12_ deficiency.

## Data Availability Statement

The raw data supporting the conclusions of this article will be made available by the authors, without undue reservation.

## Ethics Statement

The studies involving human participants were reviewed and approved by the Medical Ethics Committee of the Chinese University of Hong Kong and New Territories East Cluster of Hospital Authority of Hong Kong. The patients/participants provided their written informed consent to participate in this study.

## Author Contributions

KL, YD, and TK: conceptualization. YD and TK: data curation. KL, TZ, and XW: formal analysis. WW, RL, XW, and LL: investigation. KL, TZ, XW, and YD: methodology. TK: project administration. TK and KX: resources. TZ and KL: software. YD: supervision. KL and YD: writing—original draft. KL and XW: writing—review and editing. All authors contributed to the article and approved the submitted version.

## Conflict of Interest

The authors declare that the research was conducted in the absence of any commercial or financial relationships that could be construed as a potential conflict of interest.

## Publisher’s Note

All claims expressed in this article are solely those of the authors and do not necessarily represent those of their affiliated organizations, or those of the publisher, the editors and the reviewers. Any product that may be evaluated in this article, or claim that may be made by its manufacturer, is not guaranteed or endorsed by the publisher.

## Footnotes

1
http://www.fmrib.ox.ac.uk/fsl/
