# Supplementary material for: Cortical Short-Range Fiber Connectivity and Its Association With Deep Brain White Matter Hyperintensities in Older Diabetic People With Low Serum Vitamin B12
Source: Front Aging Neurosci. 2022 Mar 25;14:754997. doi: 10.3389/fnagi.2022.754997 (PMC8990772; doi:10.3389/fnagi.2022.754997)
Supplement: Supplementary file 1 [file Data_Sheet_1.pdf]

Supplementary Figures

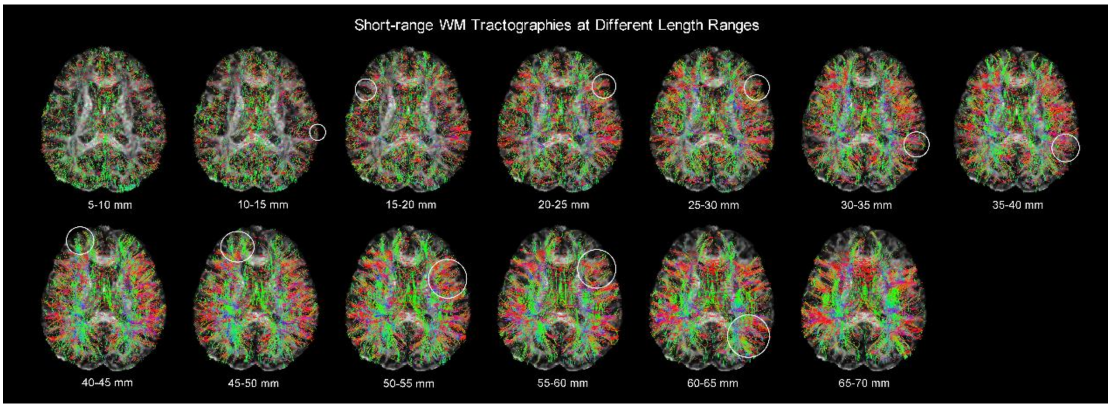

Figure S1. Short-range white matter (WM) fiber tractographies thresholded at a spectrum of fiber length constraints. Typical cortical U-fibers are illustrated with white circles. Typical U-fibers appear at the constraint of 10-15mm, while mostly disappear over 65 mm.

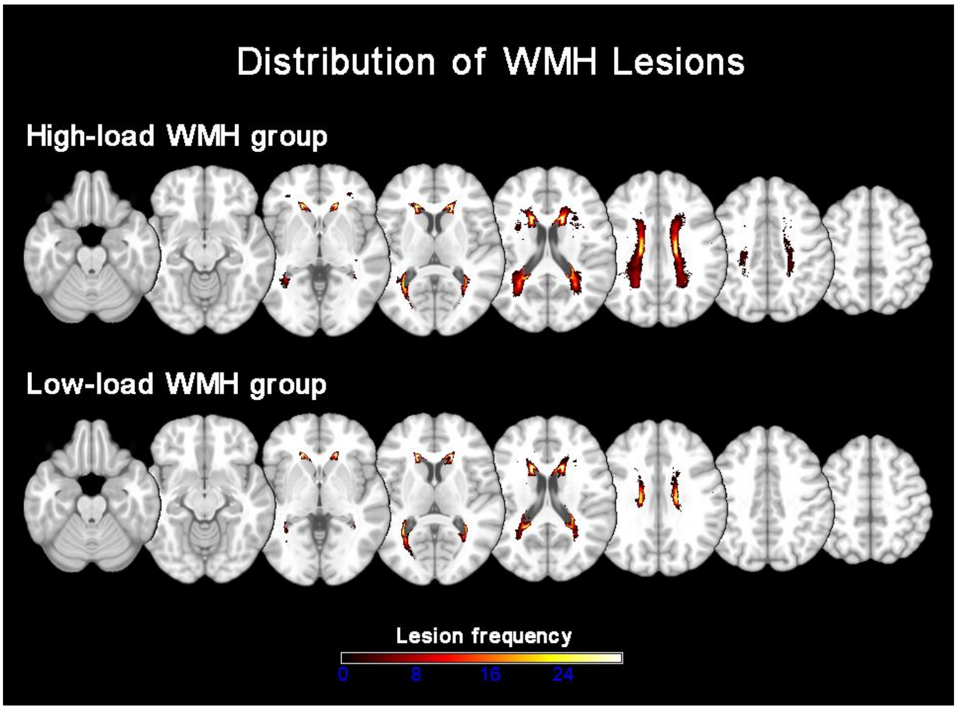

Figure S2. Unbiased automatic segmentations of white matter hyperintensities (WMHs) from each participant overlaid on a standard brain template. The color bar is coded by the lesion frequency in each voxel in the high-load and low-load DWMH groups. Visually, the high-load DWMH group showed a more widespread distribution of lesions than the low-load DWMH group.

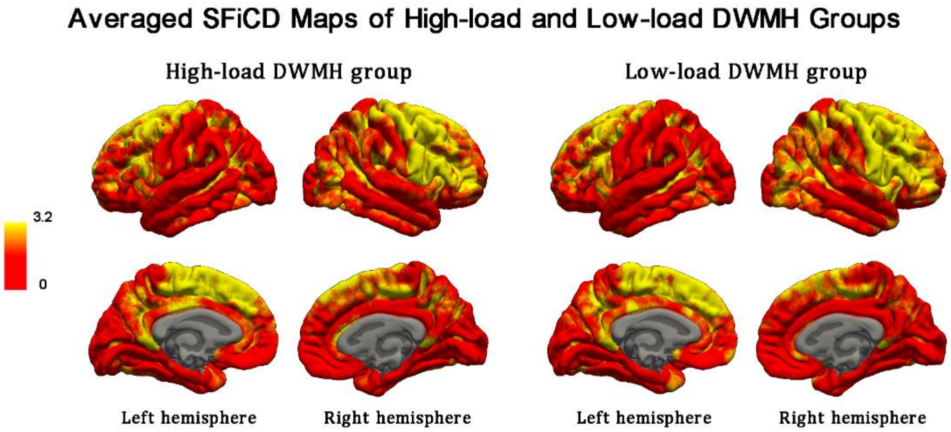

Figure S3. Averaged cortical SFiCD maps of high-load and low-load DWMH groups were shown. Generally, the prefrontal cortices, occipital cortices, posterior cingulate gyrus, precuneus, etc. show high short-range connectivity.

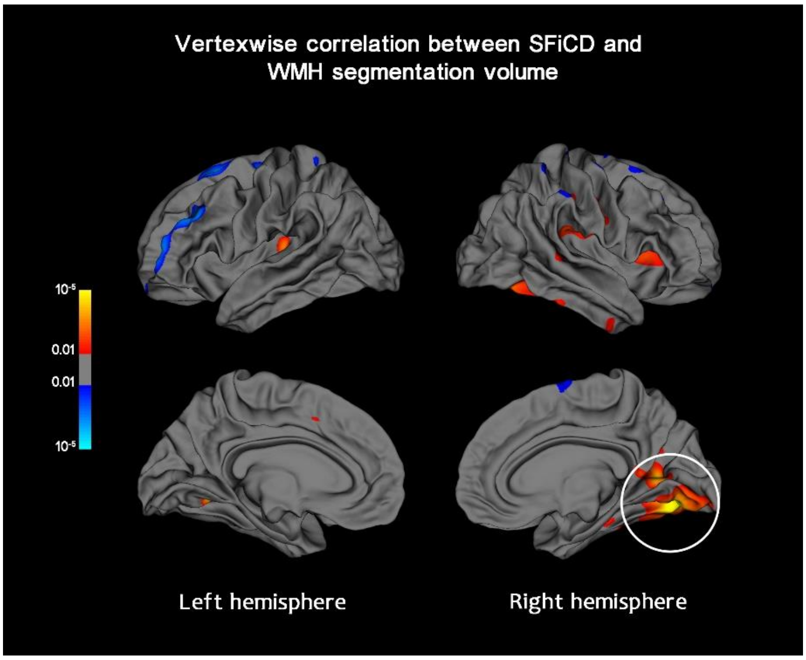

Figure S4. Vertexwise correlation between cortical SFiCD and WMH segmentation volume estimated by FreeSurfer. The statistical map is thresholded with a vertex-level *P* <0.01. Cortical regions of right fusiform gyrus and lingual gyrus (i.e., significant clusters in comparison between low-load and high-load DWMH groups) show significant correlation.

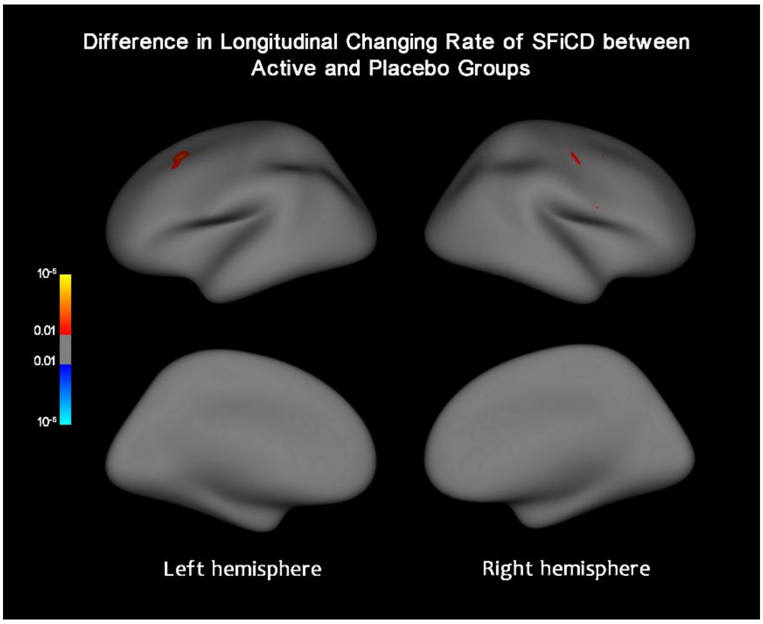

Figure S5. Vertexwise analysis of the difference in longitudinal changing rate of SFiCD between active intervention and placebo groups during the 27-month follow-up. The statistical map is thresholded with a vertex-level *P* <0.01. No cluster survives the Monte Carlo simulation correction (cluster-level *P* <0.01).

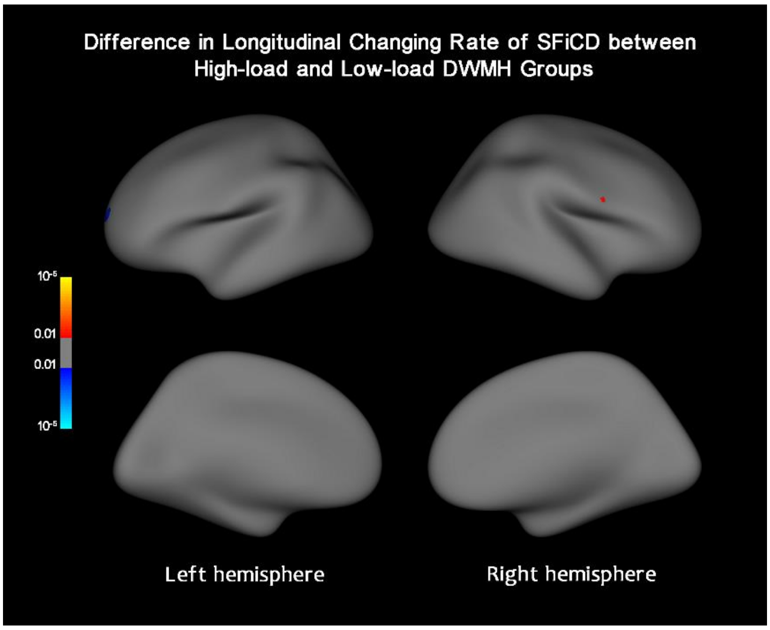

Figure S6. Vertexwise analysis of the difference in longitudinal changing rate of SFiCD between high-load and low-load DWMH groups during the 27-month follow-up. The statistical map is thresholded with a vertex-level *P* <0.01. No cluster survives the Monte Carlo simulation correction (cluster-level *P* <0.01).
