# Supplementary material for: Cortical Short-Range Fiber Connectivity and Its Association With Deep Brain White Matter Hyperintensities in Older Diabetic People With Low Serum Vitamin B12
Source: Front Aging Neurosci. 2022 Mar 25;14:754997. doi: 10.3389/fnagi.2022.754997 (PMC8990772; doi:10.3389/fnagi.2022.754997)
Supplement: Supplementary file 2 [file Data_Sheet_2.pdf]

Supplementary Tables

Table S1. Partial correlations of extracted mean short-range fiber connectivity density (SFiCD) from cortices with significant between-group difference to the scores of clinical dementia rating scale (CDR) and Neuropsychological Test Battery (NTB)

Cortical region	Fusiform gyrus		Lingual gyrus	
	r	*P*	*r*	*P*
CDR (sum of boxes)	0.036	0.797	-0.107	0.443
NTB (executive)	-0.321	0.018*	-0.265	0.053
NTB (psychomotor speed)	-0.075	0.590	-0.123	0.376
NTB (memory)	-0.110	0.429	-0.115	0.407

* stands for having statistical significance (P <0.05); NTB are counted as Z-scores by comparing to mean value, higher score indicating better performance.

Table S2. Comparison of demographic data between active intervention and placebo groups in the randomized placebo-controlled trial

	Active group (n = 30)	Placebo group (n = 29)	Statistics	P-value
Age (yrs)	73.5 (70 - 83)	73 (69 - 83)	Z = -0.480	0.631
Sex (female/male)	9/21	14/15	χ^2 = 2.071	0.150
Education (yrs)	5.25 (0 - 18.5)	5 (0 - 15)	Z = -0.426	0.670
Total intracranial volume ($\times 10^5$ mm^3)	1.49 ± 0.13	1.45 ± 0.19	T = -0.906	0.368
Cerebral white matter volume ($\times 10^5$ mm^3)	3.77 (1.40 - 4.88)	3.87 (2.69 - 5.04)	Z = -0.227	0.820
WMH segmentation volume ($\times 10^3$ mm^3)	4.97 (1.93 - 40.04)	4.04 (1.06 - 43.98)	Z = -0.197	0.844
CDR sum	0.5 (0 - 3)	0.5 (0 - 2.5)	Z = -0.523	0.601
Executive function	-0.01 ± 0.96	0.37 ± 0.72	T = 1.701	0.094
Psychomotor speed	0.03 ± 0.80	-0.08 ± 0.76	T = -0.477	0.636
Memory	0.05 ± 0.84	0.38 ± 0.76	T = 1.589	0.118
MMA at baseline (μmol/L)	0.26 (0.11 - 0.82)	0.18 (0.10 - 0.68)	Z = -1.532	0.131
MMA at month27 (μmol/L)	0.16 (0.10 - 0.28)	0.20 (0.10 - 0.69)	Z = -1.998	0.046*
Longitudinal MMA decrease (μmol/L)	0.07 (-0.01 - 0.61)	-0.02 (-0.19 - 0.19)	Z = -4.123	<0.001*
Homocysteine at baseline (μmol/L)	17.38 (8.41 - 50.92)	15.86 (10.07 - 36.77)	Z = -1.274	0.203
Homocysteine at month27 (μmol/L)	11.19 (4.11 - 11.73)	14.15 (5.98 - 34.63)	Z = -3.214	0.001*
Longitudinal homocysteine decrease (μmol/L)	5.73 (0.54 - 39.19)	0.93 (-12.02 - 18.62)	Z = -4.109	<0.001*

*: statistically significant; normally and nonnormally distributed data are described as the mean ± standard deviation and median (range), respectively; CDR = clinical dementia rating; MMA = Methylmalonic acid.

Table S3. Comparison of demographic data between high-load and low-load deep white matter hyperintensity (DWMH) groups included for the longitudinal analysis

	High-load DWMH group (n = 27)	Low-load DWMH group (n = 32)	Statistics	P-value
Age (yrs)	73 (69 - 83)	73.5 (69 - 83)	Z = -0.306	0.760
Sex (female/male)	11/16	12/20	χ^2 = 0.065	0.799
Education (yrs)	6 (0 - 15)	4 (0 - 18.5)	Z = -1.634	0.102
Total intracranial volume ($\times 10^5$ mm^3)	14.81 (11.52 - 17.79)	14.79 (10.00 - 18.10)	Z = -0.487	0.626
Cerebral white matter volume ($\times 10^5$ mm^3)	3.83 (1.40 - 5.04)	3.80 (1.71 - 4.58)	Z = -0.304	0.761
WMH segmentation volume ($\times 10^3$ mm^3)	8.20 (3.45 - 43.98)	3.08 (1.06 - 10.13)	Z = -5.051	<0.001*
CDR sum	0.5 (0 - 2.5)	0.5 (0 - 3)	Z = -0.111	0.911
Executive function	0.16 ± 0.77	0.20 ± 0.95	T = 0.158	0.875
Psychomotor speed	-0.07 ± 0.85	0.01 ± 0.72	T = 0.393	0.696
Memory	0.22 ± 0.71	0.22 ± 0.91	T = -0.001	0.999

*: statistically significant; normally and nonnormally distributed data are described as the mean ± standard deviation and median (range), respectively; CDR = clinical dementia rating.

Table S4. Assessment of the relationships between confounding factors and extracted mean short-range fiber connectivity density (SFiCD) from significant cortices

Region / Confounder	Fusiform gyrus	Lingual gyrus	Temporal cortices	Left frontal cortex	Right frontal cortex
Age	*r*=0.118/ *P*=0.332	*r*=-0.091/ *P*=0.453	*r*=-0.228/ *P*=0.058	*r*=-0.059/ *P*=0.656	*r*=-0.021/ *P*=0.873
Education	*r*=-0.043/ *P*=0.723	*r*=0.303/ *P*=0.011*	*r*=0.119/ *P*=0.326	*r*=-0.112/ *P*=0.397	*r*=-0.011/ *P*=0.936
Total intracranial volume	*r*=-0.028/ *P*=0.817	*r*=0.196/ *P*=0.105	*r*=0.158/ *P*=0.193	*r*=0.001/ *P*=0.993	*r*=-0.065/ *P*=0.623
Sex	*Z*=-0.133/ *P*=0.910	*Z*=-1.359/ *P*=0.174	*T*=0.977/ *P*=0.332	*Z*=-0.062/ *P*=0.950	*Z*=-0.443/ *P*=0.658
Metformin use	*Z*=-1.276/ *P*=0.202	*Z*=-0.747/ *P*=0.455	*T*=-1.748/ *P*=0.085	*Z*=-1.330/ *P*=0.184	*Z*=-0.996/ *P*=0.334
Aspirin use	*Z*=-1.317/ *P*=0.188	*Z*=-1.465/ *P*=0.143	*T*=-0.752/ *P*=0.454	*Z*=-0.210/ *P*=0.833	*Z*=-0.487/ *P*=0.626

* stands for having statistical significance (P <0.05); For quantitative variables, r is used to describe correlations; For dichotomous variables, T and Z are used to describe between-group comparisons respectively for normally and nonnormally distributed data.
