# Supplementary material for: Cortical Short-Range Fiber Connectivity and Its Association With Deep Brain White Matter Hyperintensities in Older Diabetic People With Low Serum Vitamin B12
Source: Front Aging Neurosci. 2022 Mar 25;14:754997. doi: 10.3389/fnagi.2022.754997 (PMC8990772; doi:10.3389/fnagi.2022.754997)
Supplement: Supplementary file 3 [file Data_Sheet_3.pdf]

Cortical Short-Range Fiber Connectivity and its Association with Deep Brain White Matter Hyperintensities in Older Diabetic People with Low Serum Vitamin B_{12}

Supplementary Appendix

Preprocessing of 3D-T1WI

Preprocessing of 3D-T1WI anatomical images followed the standard pipeline of FreeSurfer (version 6.0.0, surfer.nmr.mgh.harvard.edu). Major preprocessing steps included intensity inhomogeneity correction, removal of nonbrain tissue, automated Talairach transformation, and brain tissue segmentation(Fischl et al., 2002). For cortical construction, the border between the cortex and subcortical WM was tessellated and then topologically corrected. Finally, surface deformation was performed based on the largest shift of intensity gradients to locate the grey matter (GM)-WM and GM-cerebrospinal fluid boundaries(Fischl et al., 1999). With the above procedures, the GM-WM interface was constructed and utilized as seeds for fiber tracking in the following steps.

For the longitudinal analysis, repeated MR data were preprocessed using the FreeSurfer longitudinal pipeline(Reuter et al., 2012). Briefly, an unbiased within-subject template (i.e., base) was constructed using robust, inversely consistent registration(Reuter et al., 2010) based on the scans of two time points. Then, skull

stripping, Talairach transforms, and surface maps were initialized with common information from the within-subject template. Finally, longitudinal time-points were constructed from the bases.

The processed images by FreeSurfer pipeline were visually inspected for errors in preprocessing or segmentation, and if needed, edits were made to correct misidentified regions.

Results of validation analysis

First, for the assessment of the relationships between resulting SFiCD values and confounding factors, only one significant correlation was found between education level and SFiCD in the right lingual gyrus (Table S4). Meanwhile, it was noted that the result of higher SFiCD in the right lingual gyrus in ***Results 3.2*** was drawn with education added as covariate. Therefore, we believe that the finding in the right lingual gyrus will not be biased by education.

Second, for the possible impact of dynamic change in WMH volume during 27-month follow-up, we found it was not significantly correlated to the longitudinal SFiCD change in either the left (r =0.073, P =0.584) or the right (r =-0.017, P =0.896) middle-inferior frontal cortex. In addition, the correlations remained insignificant after controlling for age, sex, education, and total intracranial volume (Left: r =0.112, P =0.417; Right: r =0.040, P =0.772).

Third, specially for the longitudinal part, two linear regressions were performed respectively with longitudinal SFiCD changes in the left and right middle-inferior

frontal cortices added as dependent variables. Here, backward linear regression model was used, with the significance level for removal being 0.10. Finally, both of the two regression models did not reach statistical significance. For the left middle-inferior frontal cortex, a model of "SFiCD = 0.044 - 0.015*VB_{12} supplementation - 0.017*metformin" was found with highest adjusted R^2 of 0.047 (P =0.098). Here, P-values for the two independent variables of VB_{12} supplementation and metformin use were respectively 0.078 and 0.210. For the right middle-inferior frontal cortex, a model of "SFiCD = 0.162 - 0.017*sex - 0.007*total intracranial volume - 0.02*metformin" was found with highest adjusted R^2 of 0.033 (P =0.186). Here, P-values for the three independent variables of sex, total intracranial volume, and metformin use were respectively 0.200, 0.100, and 0.226.

Fourth, to further confirm that the longitudinal SFiCD increase in the bilateral frontal cortices was not driven by the wrinkling effect of brain cortical atrophy during 27 months (i.e., a relatively increased number of fibers per unit area), the correlation between longitudinal SFiCD change and change in cortical surface area was assessed. Finally, results showed insignificant correlations for the left (r =0.028, P =0.834) and right (r =-0.023 and P =0.863) middle-inferior frontal cortices.

References

Fischl, B., Salat, D.H., Busa, E., Albert, M., Dieterich, M., Haselgrove, C., van der Kouwe, A., Killiany, R., Kennedy, D., Klaveness, S., Montillo, A., Makris, N., Rosen,

B., Dale, A.M., 2002. Whole brain segmentation: automated labeling of neuroanatomical structures in the human brain. Neuron 33, 341-355.
Fischl, B., Sereno, M.I., Dale, A.M., 1999. Cortical surface-based analysis. II: Inflation, flattening, and a surface-based coordinate system. Neuroimage 9, 195-207.
Reuter, M., Rosas, H.D., Fischl, B., 2010. Highly accurate inverse consistent registration: a robust approach. Neuroimage 53, 1181-1196.
Reuter, M., Schmansky, N.J., Rosas, H.D., Fischl, B., 2012. Within-subject template estimation for unbiased longitudinal image analysis. Neuroimage 61, 1402-1418.
